# Supplementary material for: Reviving the Weizmann process for commercial n-butanol production
Source: Nat Commun. 2018 Sep 11;9:3682. doi: 10.1038/s41467-018-05661-z (PMC6134114; doi:10.1038/s41467-018-05661-z)
Supplement: Supplementary file 1 — Supplementary Information [file 41467_2018_5661_MOESM1_ESM.docx]

Reviving the Weizmann process for commercial *n*-butanol production

Nguyen *et al.*

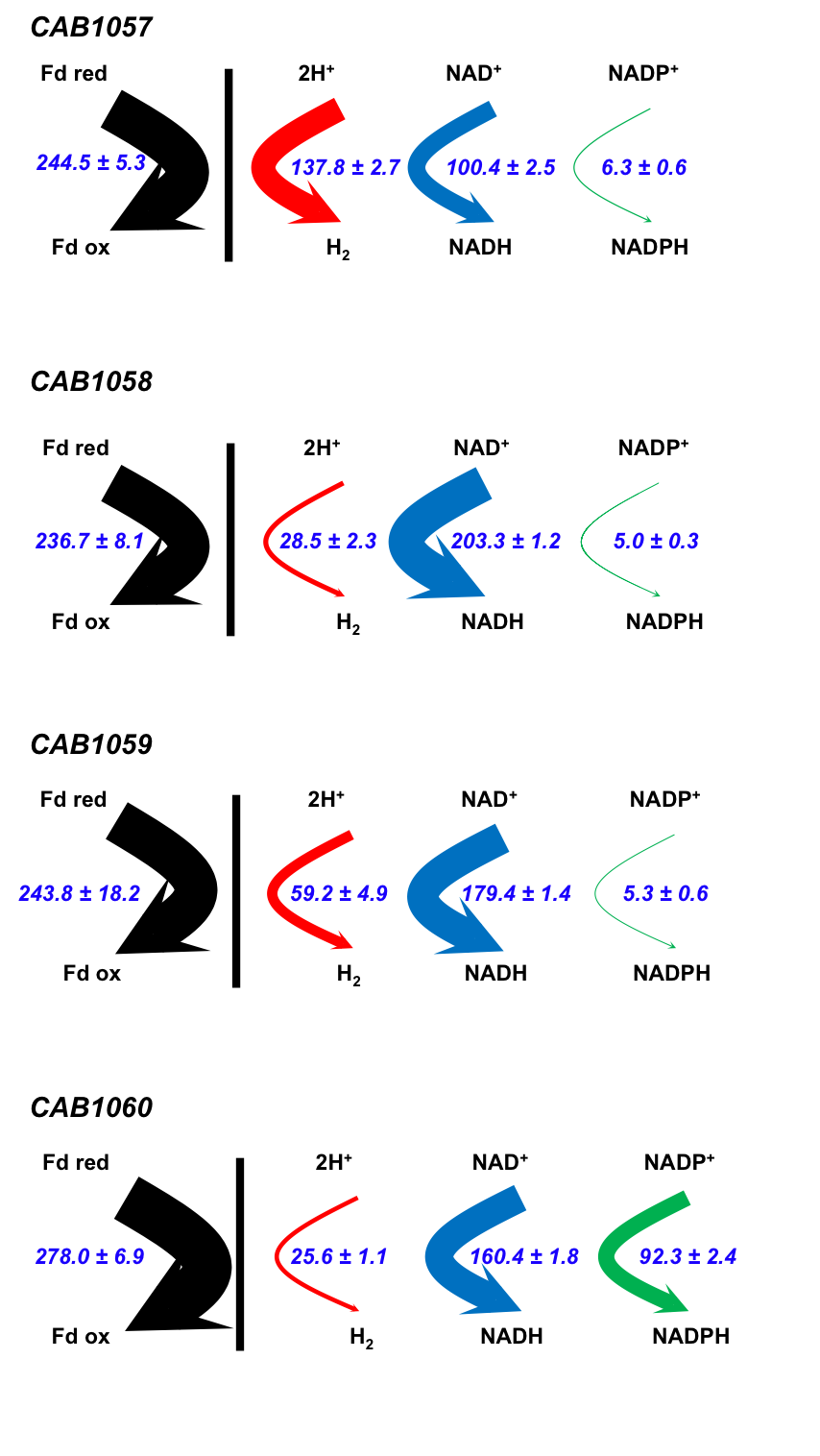


**Supplementary Figure 1.** Metabolic flux analysis of the different n-butanol producing engineered strains. All strains were evaluated in phosphate limited chemostat cultures at pH 5.0. All values (mmol/gDCW/h) are normalized to the flux of glucose consumption. No acetone, lactate and butyrate were produced by the different recombinant strains.

**Supplementary Figure 2.** Enzymatic assays on crude extracts of different *C. acetobutylicum* mutants. All strains were evaluated in phosphate limited chemostat cultures at pH 5.0.

**(A)**

**(B)**

**(C)**

**Supplementary Figure 3.** Phosphate limited high cell density continuous cultures of C. acetobutylicum CAB1060. (A) Concentration of glucose, acetate, ethanol and n-butanol in the fermentor. (B) Concentration of glucose, acetate, ethanol and n-butanol in the boiler. (C) Concentration of acetate, ethanol and n-butanol in the solvent rich phase of the decanter.

**Supplementary Table 1.** Performences of the three runs of continuous extractive fermentation

| **Run** | **Duration of the fed-batch phase (h)** | **Time steady state reached (h)** | **Butanol productivity at steady state**  **(g.l^-1^.h^-1^)** | **Butanol yield at steady state (g.g^-1^)** |
| --- | --- | --- | --- | --- |
| *I* | 81 | 103 | 14.5 | 0.34 |
| *II* | 58 | 93 | 13.4 | 0.35 |
| *III* | 69 | 82 | 13.8 | 0.35 |

**Supplementary Table 2.** List of strains

| **Strain** | **Relevant characteristics** | **Source** |
| --- | --- | --- |
| *Clostridium acetobutylicum* ATCC824 | Wild type | ATCC |
| *Clostridium kluyveri* DSM555 |  | DSMZ |
| MGCΔcac1502Δupp | ΔCAC 1502*Δupp* | (*13*, *16*) |
| MGCΔcac1502Δupp*ΔptbΔbuk* | ΔCAC1502*ΔuppΔptbΔbuk* | (*12*) |
| CAB1057 | *Δ*CAC1502*ΔuppΔptbΔbukΔ*ctfAB*ΔldhA* | This study |
| CAB1058 | *Δ*CAC1502*ΔuppΔptbΔbukΔ*ctfAB*ΔldhAΔrexA* | This study |
| CAB1059 | *Δ*CAC1502*ΔuppΔptbΔbukΔ*ctfAB*ΔldhAΔrexAΔthlA::atoB* | This study |
| CAB1060 | *Δ*CAC1502*ΔuppΔptbΔbukΔ*ctfAB*ΔldhAΔrexA ΔthlA::atoB Δhbd::hbd1* | This study |

**Supplementary Table 3.** List of plasmids

| **Plasmid** | **Characteristics** | **Source** |
| --- | --- | --- |
| pCLF1 | Cm^r^ FLP | (*13*) |
| pSOS95 | Ap^r^ MLS^r^, repL, ColE1, acetone operon | (*31*) |
| pSOS95-atoBs | Ap^r^ MLS^r^, repL, ColE1*,* *atoB* operon | (*22*) |
| pREPctfAB::upp | Cm^r^ MLS^r^ upp ΔctfAB | (*13*) |
| pREPldhA::upp | Cm^r^ MLS^r^ upp ΔldhA | (*13*) |
| pEryUpp-atoB | MLS^r^ *upp ΔthlA atoB* | This study, (*22*) |
| pEryUpp-thlA | MLS^r^ *upp ΔthlA* | (*22*) |
| pSOS95atoBs | MLS^r^ *upp atoB* | (*22*) |
| pSOS95-upp-hbd1-catP-oriRepA | Ap^r^ MLS^r^, *Δhbd::hbd1*, upp, Cm^r^, *repL* gene, repA gene | This study |
| pSOS95-MLSr-upp-Δrex-catP | Ap^r^ MLS^r^, *Δrex*, upp, Cm^r^, *repL*, colE1 | This study, (*22*) |

**Supplementary Table 4.** List of primers

| **Primers** | **Sequence** |
| --- | --- |
| Primer1b | TTTTggatccgctgttttacttggacataatactgaaaaaatgtc |
| Primer2 | taaacagacctccctaattaattattagcagctttaacttgagctattaattctgg |
| Primer3 | ttagggaggtctgtttaatgagtattaaaagtgtagcggttttaggta |
| Primer4 | ttttgccggctttt*aggcct*ttaataagcgaagaatccttttcctgattttc |
| Primer5 | Tttttggccatttt*aggcct*tcaaaataagtttacaagaatccccattatca |
| Primer6 | ttttagatctttatagctgtatattattctctcttgcataataaa |
| Primer1-hbd1-sacII | aaaag*ccgcgg*tgatccgctgttttacttggacataatactgaaaaaatgtcaaagg |
| Primer6-hbd6-sacII | AAAAA*CCGCGG*TGATCTTTATAGCTGTATATTATTCTCTCTTGCATAATAAAACAAATATTGCTGAGC |
| oriRepA-XbaI-F | AAAAA*TCTAGA*GAGCTTGGCACCCAGCCTG |
| oriRepA-5'-R | GCATTGGTAACTGTCAGACCAATTCCCGACAGTAAG |
| Amp3'-F | GGTCTGACAGTTACCAATGCTTAATCAGTGAGGCACC |
| Amp-ScaI-R | TTTTTT*AGTACT*CACCAGTCACAGAAAAGCATC |
| etfB-3-F | GACAGGGAGAAGTTATTGATAAGC |
| cac2706-3-Rb | GGCTTCTTAACACATAAAAGTACC |
| catP-3D | GGAAGGAAAGCCAAATGCTCCGG |
| catP-5R | GTACAAGGTACACTTGCAAAGTAGTGG |
| Atob-1 | ACTTATGAAATAGATTGAAATGGTTTATCTGTTACCCCGTAGGATCCTCGAAGGTCGACCA |
| Atob-2 | CTAATTTATAATTCTACAGAGTTATTTTTAACAATACTTTTTAGTTTAGTCGTTCTATTACCA |
| Rex 1 | AAGGAAAAAAGCGGCCGCAAGCTTACAAAGTGCTACA CGGGTTTTTTGCCC |
| Rex 2 | CATTACCGTACTAATCTCGGCTTTTTCGCGATTTGAC |
| Rex 3 | CAAAAGTGATATTAAGTAAGAGGTGGTCAAATCGCGA AAAAGCCGAGATTAGTACGGTAATG |
| Rex 4 | AAGGAAAAAAGCGGCCGCAAATCTTTCTGTTTCATCA ATTTCTGC |
| Rex 0 | GCATCTAGGAAATATCTATCATGAG |
| Rex 5 | GAAAGAATAACTCCTGTAGTACCGC |
